# Toxic shock syndrome associated with menstrual cup use

**DOI:** 10.1016/j.idcr.2021.e01171

**Published:** 2021-05-28

**Authors:** Hind El Soufi, Yahya El Soufi, Sarah Al-Nuaimi, Farshad Bagheri

**Affiliations:** a Department of Internal Medicine, Jamaica Hospital Medical Center, Queens, NY, United States; b Lebanese University, Faculty of Medical Sciences, Beirut, Lebanon; c Director of Infectious Diseases Department, Jamaica Hospital Medical Center, Queens, NY, United States

**Keywords:** Toxic shock syndrome, *Staphylococcus aureus*, Vaginal cup, Menstrual cup

## Abstract

Staphylococcal toxic shock syndrome is a rare but life-threatening condition. It occurs when *Staphylococcus aureus* bacteria colonizing the vagina of a healthy woman, produce toxic shock syndrome toxin 1 activating the immune system and leading to multiorgan failure. Menstrual cups also known as vaginal cups are usually used as alternatives to other intravaginal products for menstrual blood collection. In rare cases, they can also lead to toxic shock syndrome. We report a case of toxic shock syndrome associated with vaginal cup use in a healthy menstruating woman. The diagnosis was made based on the United States Centers for Disease Control and Prevention criteria of the syndrome and confirmed with a vaginal cultural growth of *Staphylococcus aureus*.

## Introduction

Toxic shock syndrome (TSS) is a toxin-mediated clinical illness caused by *Staphylococcus aureus* (*S. aureus*) and *Streptococcus pyogenes* [1]. It is characterized by fever, hypotension, rash and multiorgan failure with at least three organs being involved [2]. It usually occurs in healthy menstruating women using intravaginal products such as tampons [3]. These products act like a good milieu for colonization with bacteria capable of producing toxins known as toxic shock syndrome toxin 1 (TSST-1) [2]. When these toxins gain access to the bloodstream, they can induce a systemic illness that can sometimes be life-threatening [4].

Menstrual cups used for menstrual blood collection are considered to be an acceptable substitute for tampons [5]. They are affordable, safe and effective [5]. However, they can cause serious complications when not properly sterilized before each use [6]. Despite being in the market for a long time, cups have been rarely reported as a direct cause of TSS.

We report a rare case of TSS related to menstrual cup use in a 20-year-old menstruating woman who was using cups for one year.

## Case

A 20-year-old woman was referred to the emergency department (ED) by her primary care physician, on the 6th day of menstruation, for fever, chills, vomiting and lower abdominal pain of 4 days duration. The patient has no significant past medical history and is not sexually active. Her menstrual cycles are usually regular and last three days. One year ago, she stopped using pads for menstrual blood collection and started using the reusable vaginal cups with proper cleaning hygiene between the uses. However, because she ran out of soap this time, she was just rinsing the cups with hot water before each use. The menstrual cup was removed by the patient before presentation to the hospital. Upon arrival to the ED, the vitals were significant for hypotension (blood pressure of 96/60 mmHg), tachycardia (heart rate of 156 beats/min), fever (Temperature of 102.8 F), respiratory rate of 18 breaths per minute and a 100 % oxygen saturation on room air. On physical examination, the abdomen was soft, but tender to palpation. Upon inspection, an erythematous rash was seen on the lower abdomen. Vaginal examination revealed swelling and erythema of the labia majora bilaterally with yellowish vaginal discharge and mild menstrual spotting. No cervical motion tenderness was present. Blood tests were significant for leukocytosis with white blood cells of 15.7 K/uL (ref 4.8–10.8 K/uL), neutrophilia of 93.8 % (ref 44–80 %), bandemia of 39 % (ref 2–10 %), lactic acidosis of 2.16 mmol/L (ref 0−2 mmol/L), and elevated procalcitonin of 8.68 ng/mL (ref 0−0.09 ng/mL). Cultures from the vagina, urine and blood were obtained, and the patient was started on intravenous antibiotics including piperacillin-tazobactam, vancomycin and clindamycin. Chlamydia, gonorrhea, and HIV tests came back negative. Despite aggressive fluid resuscitation and broad-spectrum antibiotics, the patient remained hypotensive for the next few hours. She was then started on vasopressors and was admitted to the intensive care unit (ICU). Infectious diseases services were consulted, and a dose of intravenous immunoglobulin (IVIG) was given due to the deterioration of her clinical condition. On the second day of hospitalization, the genital culture grew *S. aureus* and antibiotics were then de-escalated to clindamycin and vancomycin. Her condition started to improve afterwards, and the decision was made to not continue with IVIG therapy. On day three of admission, the patient was off vasopressors. Follow up of the genital culture showed Methicillin-susceptible *S. aureus* (MSSA) clindamycin-resistant and antibiotics were then switched to cefazolin. Patient remained clinically stable and was subsequently discharged home with an outpatient follow-up plan.

## Discussion

TSS is a rare but severe condition that can lead to multi-organ failure [1]. It is mostly caused by toxins produced by *S. aureus* bacteria; however, it can also be caused by toxins produced by Group A *Streptococcus*. More specifically, most reported cases of TSS have been due to MSSA but patients infected with methicillin resistant *S. aureus* (MRSA) may also develop TSS [2,3]. TSST-1 was the first exotoxin isolated from S. aureus isolates causing TSS. These exotoxins act like superantigens capable of activating T cells in the body bypassing the antigen-presenting cells and directly interacting with the invariant region of the class II MHC molecules [2]. TSS most often occur in menstruating women aged 15–25; however, it can also occur in all ages including non-menstruating women, elderly and even children. In fact, the first few cases described of the syndrome associated with *S. aureus* were pediatric cases [7]. In non-menstrual TSS cases which represent almost half of the overall cases, risk factors can include postpartum wound infection, complicated cutaneous lesions and traumas, necrotizing fasciitis, viral infection such as Varicella and Influenza, pharyngitis and even enterocolitis [8,9]. Diagnosis for TSS is usually based on the United States Centers for Disease Control and Prevention (CDC) clinical criteria which include fever, hypotension, diffuse erythroderma, and involvement of at least three organ systems, in addition to negative cultures for alternative pathogens and negative serologic tests for other conditions [5]. In previously published reports, most cases of TSS were related to the use of tampons with high absorbency, the continuous use of tampons for more days of the menses, and the use of a single tampon for a longer period of time [10,11]. Menstrual cups use is also proven to cause TSS.

Menstrual cups are sometimes used as alternatives to other menstrual products. They are inserted into the vagina and can hold up to 40 mL of blood [12]. They should usually be emptied every four to twelve hours depending on menstrual flow and the type of the cup [12]. Menstrual cups are made of medical-grade silicone, rubber, latex, or elastomer and can last for up to ten years [12].

A study published by Nonfoux et al. in 2018 aimed to evaluate the impact of currently marketed tampons and menstrual cups on S. aureus growth and TSST-1 production [13]. Their effects were studied in vitro under experimental conditions close to those of typical tampon and cup usage. They found that higher levels of S. aureus growth and toxin production were seen in menstrual cups than in tampons, likely due to the additional air introduced into the bag by cups [13].

In 2015, Mitchell et al. reported the first detailed case of TSS related to menstrual cup use in a 37-year-old woman using cups for her first time [5]. The patient presented to the ED after 10 days of fever, myalgia, vaginal discharge and diffuse desquamation to thorax, thighs and perineum. Her blood and urine cultures grew MSSA and the case was successfully treated with Linezolid and the addition of clindamycin.

In August 2019, a meta-analysis tackling the safety of menstrual cup use was published by Van Eijk et al. [12]. Forty-three studies were eligible for review. Out of 3319 participants in these studies, five case reports about the association of menstrual cups with TSS were identified [12]. The reported case by Mitchell et al. was included in their systemic review.

In July 2020, Neumann et al. reported a confirmed case of TSS in a 33-year-old female using menstrual cup [6].

In a randomized controlled study analyzing the safety of menstrual cups and sanitary pads use in primary schoolgirls of rural western Kenya in which 751 girls were enrolled, no TSS cases were reported [14]. This study was published by Phillips-Howard et al. in 2016.

In our case report, we reported a rare case of menstrual cup-associated TSS in a healthy menstruating 20-year-old woman with the diagnosis confirmed based on the six criteria of the disease defined by the CDC and supported by vaginal cultural growth of MSSA.

Management of TSS includes aggressive fluid resuscitation, vasopressors if needed, in addition to broad-spectrum antibiotics [15,16]. Antibiotic therapy should include a toxin-suppressing agent such as clindamycin or linezolid with the latter sometimes used as monotherapy for its coverage of *S. aureus* and its ability to suppress the bacteria toxin production [17]. Despite not having enough clinical data about the efficacy of IVIG in the management of TSS, it has been previously used in cases where TSS is not responding to usual treatment [18]. Due to the rapid clinical worsening of our patient, a trial of IVIG treatment was added the same day of presentation. However, it was discontinued on the second day due to the significant improvement of her condition. In a previously published case report by Cone et al. in 1992, a 39-year-old, HIV positive man who presented with a diffuse erythema of the skin and a lesion, grew a TSST-1-producing S. aureus [18]. IVIG therapy was given for five days after failing antibiotic therapy and have led to significant improvement of symptoms [18].

## Funding statement

The authors received no financial support or funding for the publication of this article.

## Declaration of Competing Interest

The authors report no declarations of interest
